# Habitat and Season Drive Chigger Mite Diversity and Abundance on Small Mammals in Peninsular Malaysia

**DOI:** 10.3390/pathogens11101087

**Published:** 2022-09-23

**Authors:** Hadil Alkathiry, Ahmed Al-Rofaai, Zubaidah Ya’cob, Tamsin S. Cutmore, Siti Nurul Izzah Mohd-Azami, Nurul Aini Husin, Fang Shiang Lim, Sirikamon Koosakulnirand, Nor Hidayana Mahfodz, Siti Nabilah Ishak, Shih Keng Loong, Alexandr Stekolnikov, Farah Shafawati Mohd-Taib, Sazaly Abubakar, Benjamin L. Makepeace, Kittipong Chaisiri, Jing Jing Khoo

**Affiliations:** 1 Institute of Infection, Veterinary & Ecological Sciences, University of Liverpool, Liverpool L3 5RF, UK; 2 Department of Biology, Imam Muhammad Ibn Saud Islamic University, Riyadh 13318, Saudi Arabia; 3 Tropical Infectious Diseases Research and Education Centre, Universiti Malaya, Kuala Lumpur 50603, Malaysia; 4 Faculty of Tropical Medicine, Mahidol University, Bangkok 10400, Thailand; 5 School of Environmental Science and Natural Resources, Faculty of Science and Technology, University Kebangsaan Malaysia, Bangi 43600, Malaysia; 6 Zoological Institute, Russian Academy of Sciences, 199034 St. Petersburg, Russia

**Keywords:** scrub typhus, oil palm, tropical infection, *Rattus tanezumi*, *Tupaia glis*, trombiculid, Leptotrombidium

## Abstract

Chigger mites are vectors of the bacterial disease scrub typhus, caused by *Orientia* spp. The bacterium is vertically transmitted in the vector and horizontally transmitted to terrestrial vertebrates (primarily wild small mammals), with humans as incidental hosts. Previous studies have shown that the size of the chigger populations is correlated with the density of small mammals in scrub typhus-endemic regions. Here, we explore interactions between the small mammals and chiggers in two oil palm plantations located in the Perak and Johor states of Peninsular Malaysia. The location in Perak also contained an aboriginal (Orang Asli) settlement. A ~5% sub-sample from 40,736 chigger specimens was identified from five species of small mammals (*n* = 217), revealing 14 chigger species, including two new records for Malaysia. The abundance and species richness of chiggers were significantly affected by habitat type (highest in forest border), state (highest in Perak), and season (highest in dry). The overall prevalence of *Orientia tsutsugamushi* DNA in small-mammal tissues was 11.7% and was not significantly affected by host or habitat characteristics, but in Johor, was positively associated with infestation by *Leptotrombidium arenicola*. These findings highlight the risk of contracting scrub typhus in oil palm plantations and associated human settlements.

## 1. Introduction

Trombiculid mites are a highly diverse and globally distributed group of ectoparasites, with more than 3000 species described worldwide [1]. Unusually among disease vectors, only the larval stage (“chigger”) is parasitic, while the active deutonymphs and adults are free-living predators that reside in soil or leaf litter. The host specificity of many chigger species is low; for instance, some species can be found on both mammalian and avian hosts, and may even parasitise other invertebrates [2]. Several species will attack humans and domestic animals, especially dogs and cats, causing a dermatosis (trombiculiasis or “scrub itch”) that can be severe in atopic hosts [3]. However, the greatest public health impact of chiggers is as vectors of scrub typhus; a disease that usually manifests as severe fever, but which can progress to potentially fatal complications if not promptly treated with appropriate antibiotics [4]. The aetiological agents of scrub typhus are intracellular bacteria of the genus *Orientia* (Rickettsiales: Rickettsiceae): *Orientia tsutsugamushi* is widely distributed across the Asia-Pacific region, while other species circulate in the Middle East and Africa, as well as South America (reviewed in Richards and Jiang [5]). The main hosts for chiggers are small mammals and ground-nesting birds, both of which can become infected with *Orientia* spp. and sustain rickettsiaemia intermittently over weeks or even months (reviewed in Elliott et al. [6]). However, laboratory studies suggest that while chiggers are readily infected with *O. tsutsugamushi* during feeding on infected rodents and can transfer the bacteria transstadially, successful transovarial transmission into the F1 generation after a horizontal transmission event is very rare [7]. Instead, chiggers appear to be infected mainly through inheritance of the infection via the maternal line and maintenance across multiple generations.

During World War II, devastating scrub typhus outbreaks occurred among both Allied and Axis armed forces due to the mass movement of people with no past exposure to the disease into rural tropical environments where the vectors and disease agents aggregated [8]. This precipitated a period of intense research on scrub typhus in the final years of the war and subsequent quarter-century, centred especially in Kuala Lumpur, Malaysia. Here, J. Ralph Audy, Robert Traub, and collaborators published prolifically while stationed at the Institute for Medical Research. Their work on the ecology of scrub typhus established the foundational paradigms that shape our understanding of the epidemiological drivers of the disease to this day [9,10]. Among these was that *Leptotrombidium* spp. chiggers are the only confirmed vectors transmitting the disease to humans in the Asia-Pacific Region [11]. However, *O. tsutsugamushi* has been detected in chiggers of several other genera (e.g., *Eutrombicula*, *Walchia*, *Gahrliepia*, *Helenicula*, and *Microtrombicula*—reviewed in Elliott, Pearson, Dahal, Thomas, Roberts, and Newton [6]), and recently, *Herpetacarus antarctica* has been identified as a vector for *Candidatus* Orientia chiloensis to humans in Chile [12]. For *O. tsutsugamushi*, non-*Leptotrombidium* chiggers are assumed to act as intrazoonotic vectors that transmit the bacteria to wildlife, although it should be noted that some non-*Leptotrombidium* will attack humans in scrub typhus-endemic areas [9]. 

Audy, Traub, and colleagues also published extensively on the environmental conditions under which scrub typhus foci were maintained or could emerge [9,10]. While “scrub typhus” is clearly a misnomer, as it can be found in habitats as diverse as subarctic glacial moraine to sandy beaches, they did note certain patterns in disease distribution, at least in the tropics of the Asia-Pacific. Although clinical cases can occur at any time of the year, outbreaks tended to be associated with humid conditions, especially on either side of the monsoon season. “Fringe habitats” or ecotones were also identified as areas of greater risk, especially where these included areas of secondary vegetation, and the disease tended to be highly focal. However, it was not until the 21st century that provincial or nationwide analyses of scrub typhus epidemiology in countries with robust case reporting systems have been performed to identify trends in incidence objectively. For instance, in Thailand, scrub typhus in humans has been positively associated with humidity [13], rainfall, temperature, elevation, and habitat complexity [14], although correlations of human disease incidence with chigger species richness (CSR) appear to vary depending on the geographic scale [15,16]. The effects of habitat on rates of *O. tsutsugamushi* infection in chiggers and/or small mammals has shown inconsistent trends with respect to the role of forest cover versus fields or settlements [16,17,18].

Malaysia lacks a national reporting system for scrub typhus and there have been no large-scale analyses of disease risk in the country using modern statistical methods. However, focalised studies over the past century have shown a high seroprevalence of scrub typhus among agricultural workers, including those employed in oil palm or rubber plantations [19,20,21], and *Orientia* infections have been detected in chiggers and small mammals in oil palm estates [22,23]. The native indigenous people of Malaysia, known locally as the “Orang Asli”, who either maintain their nomadic lifestyle or inhabit the forests of Peninsular Malaysia, exhibit particularly high exposure to scrub typhus, with one study from the 1970s reporting a seroprevalence of >70% in deep forests [24]. Here, we investigate the impact of temperature, humidity, location, and habitat on chigger abundance and CSR in oil palm plantations in two states of Peninsular Malaysia, one of which was adjacent to an Orang Asli settlement. We also analyse the relationships between chiggers, wild small mammals, and *O. tsutsugamushi* infection in hosts across these two sites. 

## 2. Materials and Methods

### 2.1. Small-Mammal Trapping

**Figure 1:**
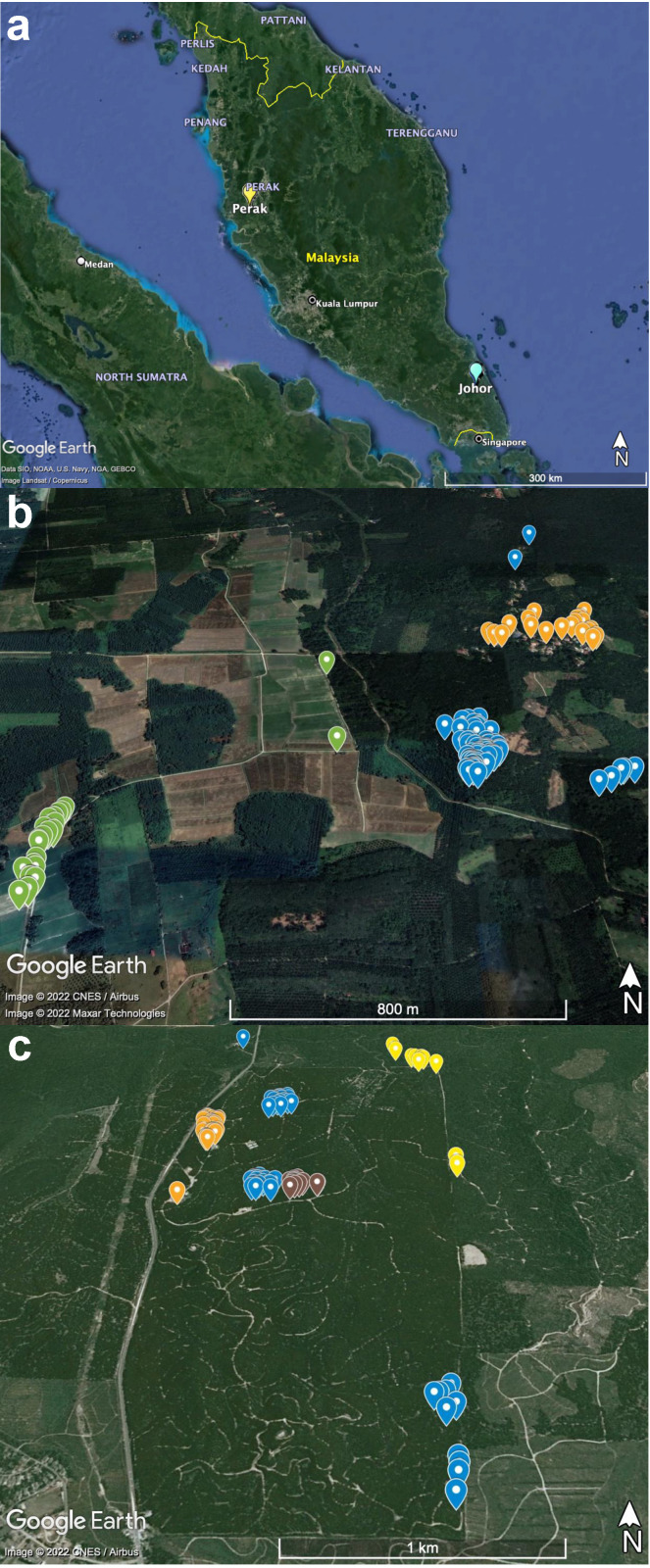
(**a**) Sample collection sites in the Perak and Johor states of Peninsular Malaysia. Locations of the small mammals captured at the (**b**) Perak and (**c**) Johor sites. Location labels are coloured according to habitat types: green—paddy field; blue—oil palm plantation; orange—human dwelling; yellow—forest border; brown—rubbish dumpsite. Google Earth maps data: (**a**) Data SIO, NOAA, U.S. Navy. NGA, GEBCO, Image Landsat/Copernicus; (**b**) Image © 2022 Maxar Technologies, (**b**,**c**) Image © 2022 CNES/Airbus.

Trapping of small mammals was performed between December 2018 and December 2019 at two separate locations: an Orang Asli village in Perak state (N4.313903, E100.929009), west-central Peninsular Malaysia, and an oil palm research plantation in Johor state, south-eastern Peninsular Malaysia (N2.02916, E103.87076) (Figure 1a). Subsequently, the sampling sites will be referred to as Perak and Johor, respectively. The Orang Asli village is situated within an oil palm plantation with paddy fields approximately 800 m distant (Figure 1b). The oil palm research plantation in Johor consists of areas designated as staff quarters, a rubbish disposal site, and a border region (delineated by a stream) to an adjacent plot of secondary forest (Figure 1c). One hundred and fifty live small-animal cage traps were used per state per season, covering an area of ~3.6 km^2^ in Perak and ~3.4 km^2^ in Johor. Baits used included corn, dried fish, and banana. Sampling in each location was conducted once in June or July, representing a dry period, and in December, representing a wet monsoon period. It is important to note that rain still occurred during the sampling periods in June/July; however, the frequency of rain was much less than during the monsoon season in December, where heavy rain was encountered daily. Each sampling period involved five trapping days with daily checking of the traps between 7:00 and 10:00 a.m. The locations of traps positive with animals, temperature, and relative humidity were recorded using an Extech RHT3: Ezsmart HygroThermometer device (Extech, New Hampshire, USA). Maps were generated from the GPS coordinates using Google Earth version 7.3.4.8573 (available at: https://www.google.com/earth/index.html, accessed on 8 August 2022). 

### 2.2. Chigger Collection

The trapped animals were euthanised humanely using carbon dioxide asphyxiation or tiletamine and zolazepam (Zoletil 100) overdose via intramuscular injection followed by cardiac puncture, and subjected to species identification based on observations of physical appearance and morphological measurements [25]. Animal tissues were collected and preserved for DNA extraction and rodent species group determination based on PCR amplification and sequencing of a mitochondrial cytochrome c oxidase I (COI) gene fragment, as previously described [26]. The rodent (*Rattus* sp.) COI sequences were deposited in the Barcode of Life Data System (BOLD) database and the process IDs are provided in Table S1. Small mammals were searched for chiggers, which were removed from the exterior of the animal carcasses and placed in 80% ethanol for transportation and long-term preservation. Chiggers from the same animals were placed in the same tube to facilitate the counting and calculation of abundance and intensity of infestation [27]. Chigger intensity (mean number of chiggers per individual) and chigger infestation rates (presence of at least one chigger on a host animal) were determined. A purposive 5% subsample from the collected chiggers from each animal was used for morphological identification, as previously described [28], to estimate species richness. 

### 2.3. Ecological Analyses

Ecological analyses were performed, as previously described, using packages implemented in the R programming environment [15,16]. Species richness was determined based on the number of chigger species observed on each host species, host sex, habitat type, collection site, and season. Five habitat types were considered in the analyses: oil palm plantation, human dwelling (including Orang Asli residences in Perak and the research and accommodation buildings within the plantation at the Johor site), paddy field, forest border, and rubbish dumpsite. The CSR and diversity estimations, including first-order Jack-knife (Jack1) and Shannon index (H’), and species accumulation curves were obtained using the “BiodiversityR” package [29]. Correspondence analysis for examining the association between chigger species and the habitat types was performed using the “FactoMineR” package [30].

### 2.4. Multiple Regression Modelling

Generalised linear models (GLM) were constructed to evaluate the contribution of the host or environmental variables to chigger abundance and species richness. Poisson regression models were generated based on chigger abundance and CSR counts using the “lme4” package [31]. Variables included in the initial models were as follows: host species, sex, maturity, habitat types, relative humidity, temperature, seasons, and state. Data distribution of the response variable (chigger abundance or CSR) was tested using the ‘descdist’ function in the ‘fitdistrplus’ package [32]. Model selection was performed based on the Akaike’s Information Criterion adjusted for small samples size (AICc) using the “glmulti” package [33]. The best model was selected from the model-averaged important terms (MaIT) >80%. The variance inflation factor was computed using a ‘vif’ function in the ‘car’ package to identify the degree of multicollinearity among the explicative variables [34]. The quality of the selected model was evaluated with goodness-of-fit using ‘chisq_gof’ functions in the ‘sjstats’ package [35].

### 2.5. Network Analyses

Bipartite network analyses, which show the degree of host or habitat specialisation among the chigger species investigated, were conducted at the community (pooled based on host species or habitats) or individual levels using the “vegan” [36] and “Bipartite” packages [37]. Bipartite network parameters, including linkage density and V-ratios, were also determined using the “Bipartite” package. Unipartite networks, which illustrate the interaction patterns within the host community based on the co-occurrences of chigger species, were obtained from the bipartite networks using the “tnet” package [38].

### 2.6. Prevalence and Co-Occurrence Analysis between Chigger Species and with O. tsutsugamushi 

DNA was extracted from the spleen of the small mammals using the NucleoSpin® Tissue Extraction Kit (Macherey-Nagel, Düren, Germany), according to the manufacturer’s protocol. The *Orientia*-specific type surface antigen 47kDa gene, *tsa47*, was used for the detection of *O. tsutsugamushi* in the samples via PCR amplification, according to a previously published protocol [39]. The ‘cooccur’ package was applied to investigate the co-occurrence of *O. tsutsugamushi* in the rodent tissues and the presence of chigger species on the corresponding rodent host using a probabilistic model of species co-occurrence [40]. This model of co-occurrence (e.g., positive, negative, or random associations) allows us to obtain the probability that paired species in a dataset co-occur at frequencies either lower or higher than the observed frequencies of co-occurrence [41]. Positive association occurs if one parasite mutually enhances the presence and/or transmission of another parasite species. In contrast, negative associations may result from direct competition between parasite species, or an environmental limitation that prevents the two species from co-occurring in a host. 

## 3. Results

### 3.1. Small-Mammal Trapping

A total of 217 animals were trapped and included in this study (Table S2). The animals were identified as Asian house rat (*Rattus tanezumi*) of R3 mitotype (*n* = 116), common treeshrew (*Tupaia glis*; *n* = 40), Malayan field rat (*Rattus tiomanicus*; *n* = 22), Polynesian rat (*Rattus exulans*; *n* = 13), ricefield rat (*Rattus argentiventer; n* = 25), and *R. tanezumi* sensu stricto (*n* = 1). Of these, 117 individuals were captured in Perak and 100 were captured in Johor. In both states, *R. tanezumi* R3 mitotype was the dominant animal species (*n^Perak^* = 64, *n^Johor^* = 52). This was followed by *R. argentiventer* in Perak (*n* = 25) and *T. glis* in Johor (*n* = 33). The majority of the animals were collected from oil palm plantations in both locations (121/217), with *R. tanezumi* R3 mitotype being the dominant animal species in plantations (78/121) followed by *T. glis* (21/121). Similar observations were made in human dwellings (47/217), where *R. tanezumi* R3 mitotype (26/47) and *T. glis* (13/47) predominated. The paddy field (26/217) was dominated by *R. argentiventer* (22/26), whereas *R. tiomanicus* and *R. exulans* were found primarily in the plantations, but also in other habitats (human dwelling and forest border). The only *R. tanezumi* sensu stricto identified was found in the paddy field. More animals were trapped during the dry season (125/217) than in the wet season (92/217). The number of males and females trapped was similar, with 103 females and 114 males, but more adults were trapped (149/217) than juveniles (65/217). Maturity level was not determined for three of the animals.

**Figure 2:**
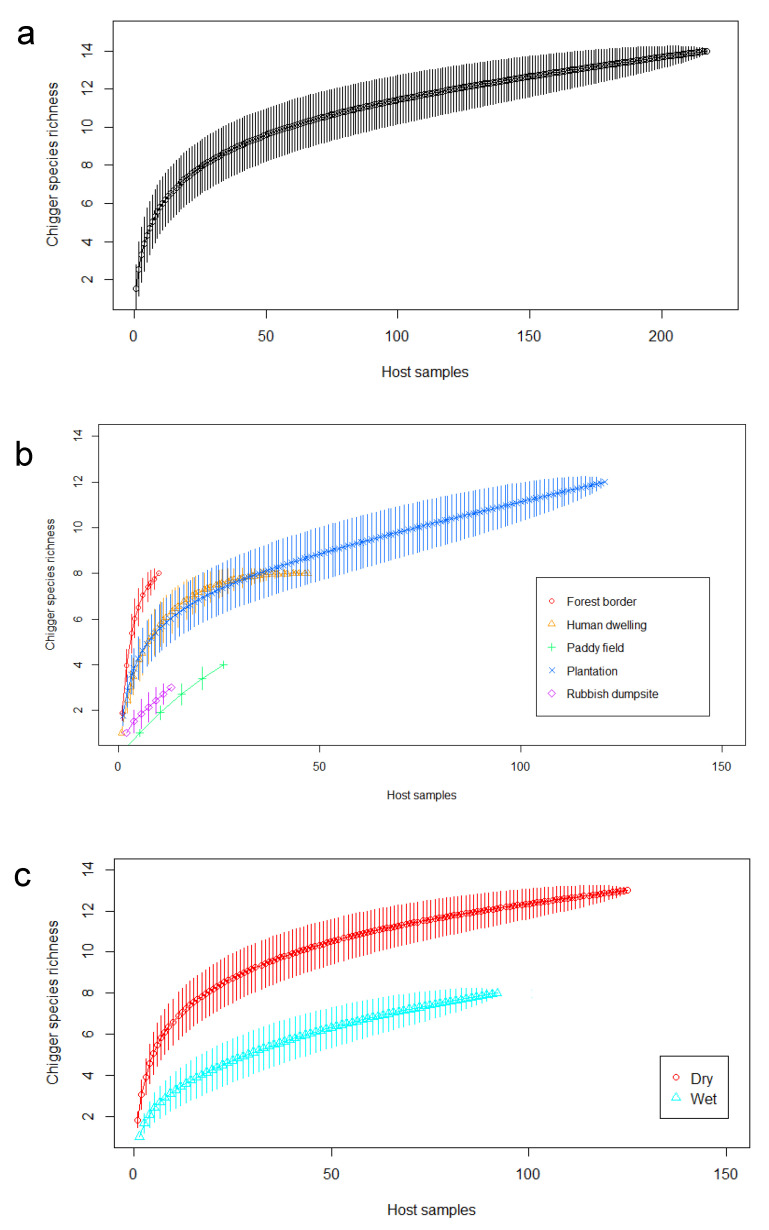
Species accumulation curve for (**a**) all collections, (**b**) collections separated by the five habitat types in the state of Perak and Johor, and (**c**) collections by season.

A species accumulation curve for the overall collection indicated that the sample size of small mammals was sufficient to describe the chigger species diversity accurately, since a plateau was reached at around 200 hosts (Figure 2a). However, observations by habitat type (Figure 2b) suggested that more sampling would be required to reveal the species richness at the forest border, rubbish dumpsite, and paddy field, as the associated curves had not plateaued at the indicated sampling size. Species accumulation curves for both wet and dry periods (Figure 2c) showed signs of saturation.

### 3.2. Chigger Fauna Identified

A total of 40,736 chiggers were collected, of which 1,954 (4.8%) were identified by subsampling, resulting in a tally of 14 species (Table 1). Three of these—*Ascoschoengastia indica*, *Leptotrombidium deliense*, and *Walchiella oudemansi*—heavily dominated the chigger fauna, accounting for >75% of the identified specimens in aggregate. Moreover, these species were also the most widespread across individual hosts, with *A. indica* and *L. deliense* attaining a prevalence of >40% each, while almost a quarter of hosts were infested with *W. oudemansi*. Two new species records for Malaysia (*Trombiculindus paniculatum* and *Walchia kritochaeta*) were identified and several other chigger species were recorded on new hosts (Table 1). 

### 3.3. Chigger Intensity and Infestation Rates

Chigger infestation status is summarised in Table S3. Of the 217 animals trapped, 169 (78%) animals were found to be infested with chiggers, with a mean chigger intensity of 241 (range = 1–2735). When host species were analysed separately, *R. tiomanicus* had the highest infestation rate (91%), followed by *T. glis* (90%), *R. tanezumi* R3 mitotype (85%), *R. exulans* (62%), and *R. argentiventer* (20%). *Rattus tanezumi* R3 mitotype had the highest mean chigger intensity (282), followed by *T. glis* (238), *R. exulans* (201), *R. tiomanicus* (117), and *R. argentiventer* (63). When all host species were considered together, chigger infestation rates were higher in the dry period (94%, mean chigger intensity = 292) than the wet period (55%, mean chigger intensity = 124). Infestation rates in females (80%) and males (76%), as well as in adults (80%) and juveniles (74%), were similar. 

Co-infestations of chigger species were observed in 45.5% (101/217) of the animal hosts (Table S4). Apart from *R. argentiventer* and *R. tanezumi* sensu stricto, in which none of the individuals was co-infested, more than 42.5% individuals from each of the other host species were found to be co-infested with at least two chigger species (Table S5). Co-infestations with two chigger species were the most common (21.1%), followed by three chigger species (18.3%; Table S4). The highest co-infestation level was recorded in a single *T. glis* juvenile male collected from the plantation at the Johor site, with five chigger species observed: *A. indica*, *Walchia lewthwaitei*, *Leptotrombidium arenicola*, *L. deliense*, and *Walchia rustica*. 

### 3.4. Influence of Host and Environmental Variables on Chigger Abundance and CSR

**Figure 3:**
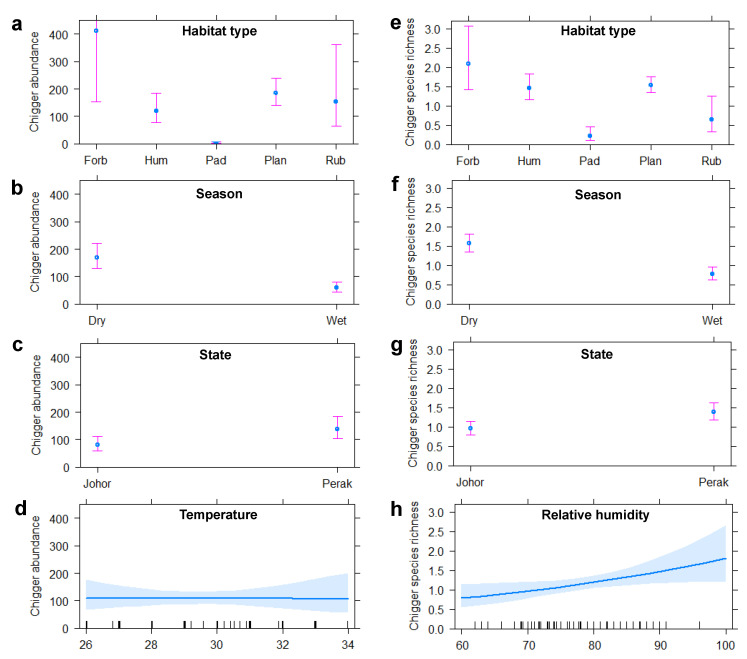
Generalised linear model effect plots for the indicated independent variables and the predicted outcomes for (**a**–**d**) chigger abundance and (**e**–**h**) species richness. Habitat types include forest border (Forb), human dwelling (Hum), paddy field (Pad), plantation (Plan), and rubbish dumpsite (Rub). Error bars represent the 95% pointwise confidence intervals of the estimated effect.

Correlation between environmental or host variables and chigger abundance and CSR were evaluated by GLMs with the best model selection based on AICc. Only animals with maturity level determined (*n* = 214) were included in the analyses from this point forward. For chigger abundance, habitat types (Chi-square = 67.220, *p* < 0.001) and seasons (Chi-square = 21.743, *p* < 0.001) were found to be significant variables in the best model (Figure S1a, Table S6). The highest level of chigger infestation on hosts was found in the forest border followed by the plantation and rubbish dumpsite (Figure 3a). Chigger abundance was lowest in human dwellings and paddy fields. No significant effect of temperature on chigger abundance was observed (Figure S1a, Table S6); whereas habitat types (Chi-square = 56.053, *p* < 0.001), seasons (Chi-square = 35.594, *p* < 0.001), and states (Chi-square = 11.933, *p* < 0.001) were identified in the best model as the variables with significant effects on CSR (Figure S1b, Table S7). The CSR was highest in forest borders followed by plantations and human dwellings, and lowest in paddy fields and the rubbish dumpsite (Figure 3e); moreover, it was considerably higher in the dry season compared to the wet season (Figure 3f). Relative humidity was not identified as a significant factor influencing CSR in the final model selection (Figure S1b, Table S7); however, there appeared to be a weak positive association (Chi-square = 5.341, *p* = 0.02, Figure 3h). Both chigger abundance (Figure 3c) and species richness (Figure 3g) were higher in Perak state. Host variables (i.e., host species, sex, and maturity) did not show significant effects.

### 3.5. Chigger Habitat Preferences 

**Figure 4:**
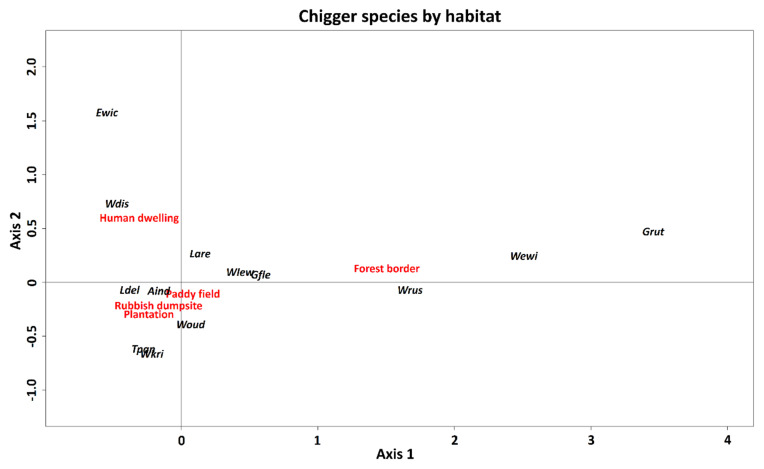
Ordination diagram of the first two axes of the canonical analysis of five habitat types (forest border, human dwelling, paddy field, plantation, and rubbish dumpsite) and 13 chigger species in Peninsular Malaysia. *Ascoschoengastia indica* (Aind), *Eutrombicula wichmanni* (Ewic), *Gahrliepia fletcheri* (Gfle), *Gahrliepia rutila* (Grut), *Leptotrombidium arenicola* (Lare), *Leptotrombidium deliense* (Ldel), *Trombiculindus paniculatum* (Tpan), *Walchia disparunguis pingue* (Wdis), *Walchia ewingi ewingi* (Wewi), *Walchia kritochaeta* (Wkri), *Walchia lewthwaitei* (Wlew), *Walchia rustica* (Wrus), and *Walchiella oudemansi* (Woud).

The ecological specialisation of some widespread chigger species with habitat types was identified using correspondence analysis, with axis 1 and axis 2 explaining ~88% of the total variance (Figure 4). Three chigger species (*Gahrliepia rutila*, *Walchia ewingi ewingi*, and *W. rustica*) showed a preference for forest border habitat whereas *A. indica*, *Eutrombicula wichmanni*, *L.* *arenicola*, *L. deliense*, *T. paniculatum*, *Walchia disparunguis pingue*, *W. kritochaeta*, and *W. oudemansi* were associated with human-modified habitats, such as the plantation, human dwelling, and rubbish dumpsite.

### 3.6. Network Analyses of Chigger–Host and Chigger–Habitat Association

**Figure 5:**
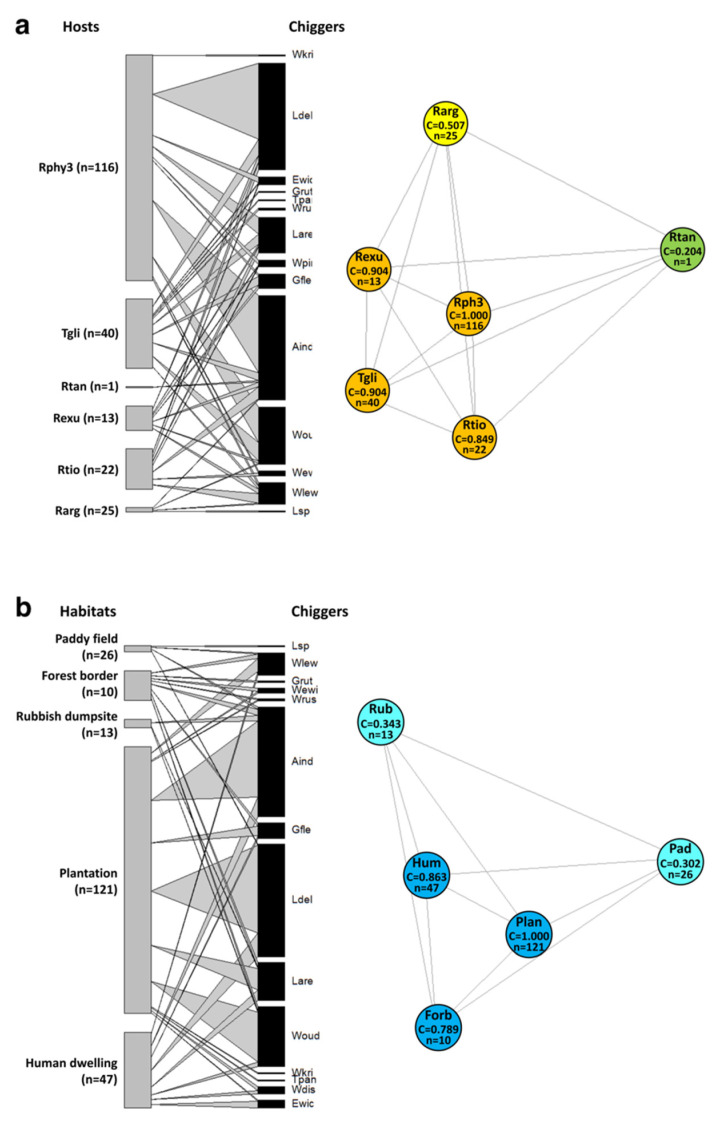
(**a**) Host- and (**b**) habitat–chigger associations in Peninsular Malaysia. (**Left** panels) Bipartite network graphs based on presence–absence data. The number of individual hosts examined is shown in parentheses. (**Right** panels) Unipartite network models and Eigenvector centrality scores (EC) based on presence–absence data, showing patterns of chigger sharing among (**a**) small-mammal host species or (**b**) habitat types. The most centrally located node has an EC closest to one. Small-mammal host species comprise *R. tanezumi* R3 mitotype (Rphy3), *T. glis* (Tgli), *R. tiomanicus* (Rtio), *R. exulans* (Rexu), *R. argentiventer* (Rarg), and *R. tanezumi* s. s. (Rtan). Habitat types comprise forest border (Forb), human dwelling (Hum), paddy field (Pad), plantation (Plan), and rubbish dumpsite (Rub). Chigger species included in the analyses: *A. indica* (Aind), *E. wichmanni* (Ewic), *G. fletcheri* (Gfle), *G. rutila* (Grut), *L.* *arenicola* (Lare), *L. deliense* (Ldel), *Leptotrombidium* sp. (Lsp), *T. paniculatum* (Tpan), *W. disparunguis pingue* (Wdis), *W. ewingi ewingi* (Wewi), *W. kritochaeta* (Wkri), *W. lewthwaitei* (Wlew), *W. rustica* (Wrus), and *W. oudemansi* (Woud).

Bipartite network analysis showed the degree of interactions of host species (Figure 5a, left panel) and habitat types (Figure 5b, left panel) with chiggers. Overall, *R. tanezumi* R3 mitotype harboured the largest chigger population, which was likely due to the sample-size bias associated with this generalist species, which was found in numerous habitat types; therefore, it had a high probability of encountering questing chiggers. The chigger population found on this species was dominated by *L*. *deliense, A. indica, W. oudemansi*, and *L. arenicola.* These chigger species also contributed to the largest chigger assemblage in the plantation habitat and, apart from *W. oudemansi*, were also common in human dwellings. More than 70% of the total chigger species discovered in this study parasitized the non-rodent host, *T. glis* (order Scandentia), which was also associated with plantations. Of these, *W. oudemansi* was the most abundant, followed by *L. deliense*; in turn, three chigger species, *W. rustica, G. rutila,* and *T. paniculatum,* parasitized *T. glis* exclusively.

One chigger species, *A. indica*, parasitized all host species in all habitat types and can therefore be considered an extreme generalist. In contrast, specialist species comprised *W*. *rustica, G. rutila*, *W. kritochaeta*, and *T. paniculatum,* which were present only on one host species and were associated with no more than two habitat types. *Rattus tanezumi* R3 mitotype and the plantation habitat were assigned the highest Eigenvector centrality scores (EC = 1.0) among all hosts (Figure 5a, right panel) and habitat types (Figure 5b, right panel), respectively, in the unipartite network models, and further supported the bipartite analysis. 

Unipartite networks generated at the individual host level based on sites and seasons provided further insights into host–chigger associations in each state (Figure S2). Although Malaysia does not experience strong seasonality, the amount of rainfall observed in different biogeographic regions of Peninsular Malaysia is highly variable. The networks for Johor (Figure S2a,b) were dominated by individuals assigned lower EC values (<0.6), regardless of season, indicating that there were fewer individuals with strong connections. Only 9/48 individuals were assigned high EC values (range 0.633–1.00) during the dry season and 2/27 individuals exhibited high EC values (1.00) during the wet season. In contrast, the networks for Perak (Figure S2c,d) displayed a higher proportion of individuals (34/62) with EC values > 0.6 (range 0.636–1.00) during the dry season, whereas only 3/17 individuals had high EC values (range 0.683–1.00) during the wet season. At Johor, the species with the highest EC shifted from *T. glis* in the dry season to *R. tanezumi* R3 mitotype and *R. tiomanicus* in the wet season. At Perak, the individual species with the highest EC shifted from *R. tanezumi* R3 mitotype and *T. glis* in the dry season to *R. tiomanicus* in the wet season. These observations suggest distinctive seasonal impacts on the host–chigger interaction patterns between the two states. This is also supported by the large differences in the linkage density and V-ratio bipartite network parameters between the dry and wet seasons in Perak, which were not apparent for Johor (Table S8). 

### 3.7. O. tsutsugamushi Prevalence and Co-Occurrence with Chigger Species and between Chigger Species

**Figure 6:**
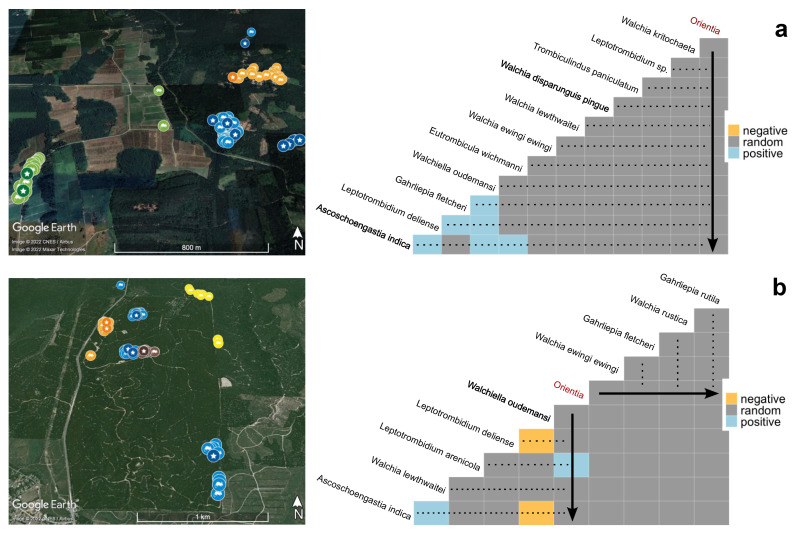
*Orientia* screening from small mammals at the (**a**) Perak and (**b**) Johor sites. Left panels: Location of the small-mammal collection points and specimens with a positive *Orientia* PCR. Location labels are coloured according to habitat types: green—paddy field; blue—oil palm plantation; orange—human dwelling; yellow—forest border; brown—rubbish dumpsite. Locations marked with stars indicate positive detection of *Orientia* sp. DNA by PCR in the spleen of the small mammal captured. Right panels: Species co-occurrence matrix for *Orientia* sp. and various chigger species detected from the small-mammal hosts. The matrix show the positive (blue), negative (yellow), or the lack of (grey, ‘random’) associations between species pairs, as determined by the probabilistic co-occurrence model based on the presence or absence of the indicated species on the animal hosts. Arrows indicate the pairwise association between *Orientia* sp. and the various chigger species along the direction of the arrows. Google Earth maps data: (**a**,**b**) Image © 2022 CNES/Airbus; (**b**) Image © 2022 Maxar Technologies.

The spleens from around 11.7% of the total small-mammal hosts examined (25/214) were positive for PCR amplification of *O*. *tsutsugamushi* DNA (Figure 6, left panels; Table S9). Excluding *R. tanezumi* sensu stricto (*n* = 1), all small-mammal species in this study showed positive *O*. *tsutsugamushi* infection, with the prevalence of infection ranging from 4.8% to 16.7%. The highest prevalence of infection was observed in *R. exulans* (16.7%) followed by *R. tanezumi* R3 mitotype (15.7%), whereas *R. tiomanicus* and *T. glis* had the lowest prevalence of infection, at 4.8% and 5.0%, respectively. Among all habitat types, plantation had the highest prevalence of infection (15.1%), while other sites showed a prevalence of less than 8.5% and none of the animal hosts from the forest border habitat were positive for *O*. *tsutsugamushi* infection. The Perak site had a higher prevalence than Johor but with an overlapping 95% CI. Infection rates with *O. tsutsugamushi* were almost identical between male and female, or adult and subadult, hosts, and there was no discernible effect of seasonality. Species co-occurrence analysis (Figure 6, right panels) identified a positive association between *O. tsutsugamushi* infection of the animal hosts and the presence of *L. arenicola* (*n* = 157) on the hosts (*n^infected^* = 10) in Johor (*p* < 0.01). However, no significant associations were observed between *O. tsutsugamushi* infection of the animal hosts and any of the chigger species found in Perak. Positive associations were also observed between several chigger species pairs in Perak: *W. oudemansi* with *G. fletcheri*, *L. deliense*, and *A. indica*; *G. fletcheri* with *L. deliense*; and *A. indica* with *L. deliense* and *E. wichmanni*. In Johor, *W. lewthwatei* and *A. indica* were positively associated with each other, whereas negative associations were apparent between *W. oudemansi* and *L. deliense,* and *W. oudemansi* and *A. indica*.

## 4. Discussion

Despite Malaysia representing an unparalleled cradle of scrub typhus research in the immediate post-World War II period, this study represents the first to apply modern statistical approaches to analyse chigger–host relationships and *Orientia* prevalence in wild animals in the country. This is timely due to renewed interest in scrub typhus in Malaysia, the importance of which is probably greatly underappreciated due to a lack of a national reporting system for the infection. In the last 10 years, only four studies have investigated scrub typhus seroprevalence or clinical cases in Malaysia, with a mean seroprevalence of 17.9% reported for the Orang Asli communities (asymptomatic individuals) across Peninsular Malaysia in 2013 [20]. A more recent febrile illness study in Sabah, East Malaysia, diagnosed scrub typhus in 27% of hospitalised patients [42]; also, a single hospitalised case was reported from Kelantan (northeast Peninsular Malaysia) in 2021, with subsequent identification of *Orientia*-positive *L. deliense* in the vicinity of the patient’s residence [43]. Earlier this year, a tertiary hospital study demonstrated a scrub typhus diagnosis in 8% (11% of those with confirmed aetiology) of febrile patients in Teluk Intan, which is only ~40 km south of our field site in Perak [44]. Underlining the potential seriousness of the disease, 4 of the 24 cases identified in Teluk Intan were classified as severe, with one death.

Compared with our previous chigger–host ecological study across Thailand, which was conducted on a much larger scale than the current work [15], both the average CSR (6.5 chigger species per 100 host individuals for Malaysia; 2.4 species per 100 hosts in Thailand) and chigger intensities (Malaysia, 241; Thailand, 42) were considerably higher in Malaysia. However, sampling in a greater diversity of habitats across Malaysia would be required to determine if this genuinely reflects national trends. From a global perspective, although the diversity of chiggers in this study was fairly high, a recent publication from China reported a staggeringly elevated chigger diversity—72 species of chiggers recovered from a single small-mammal species [45]. 

Notably, although Malaysia has one of the most intensely studied chigger faunas in the world, we discovered two new species records for the country (*T. paniculatum* and *W. kritochaeta*) and several records on new hosts [46]. *Walchia kritochaeta* was very rare in our dataset, but is of potential importance in scrub typhus epidemiology, as *Orientia*-positive specimens were recently reported from several villages in Chiang Rai province, northern Thailand [16]. Indeed, in Thailand, it has only been reported in the northern and north-eastern provinces (where an ecological preference for rainfed, flooded cultivated land was noted [15,28]); so, its occurrence in Malaysia, ~1,200 km south of these sites (especially in non-flooded plantations during the dry period), is surprising. Some chigger species exhibited ecological preferences consistent with those recorded in Thailand, such as *A. indica* aggregating in human-disturbed habitats [15], while *L. deliense* is known to be a generalist that can be abundant in habitats as diverse as villages [16], parkland [47], and forest [15]. In Thailand, *Walchia* spp. are generally associated with rainfed land and forest [15], similar to our observations in Malaysia, although *W. kritochaeta* was recently reported in proximity to human dwellings in Chiang Rai province [16]. One disparity between Thailand and Malaysia was the apparent preference of *W. d. pingue* for human dwellings, which was not the case in Thailand [15], although the sample size of this species in the current study was small.

Interestingly, we recorded a Malaysian endemic, *L. arenicola*, for the first time since the 1970s, and this species alone exhibited a positive association with *O. tsutsugamushi* infection in small mammals in the current study. Sandy beaches were reported to be the strict ecological niche of *L. arenicola* [48], which is clearly not the case, albeit it was found only at the Johor site (which is situated less than 20 km from the coast). This species has been a major focus of epidemiological and experimental studies at the Institute for Medical Research, Kuala Lumpur, where it was shown to harbour *O. tsutsugamushi* naturally and in the laboratory could transmit the pathogen horizontally to mice, as well as transovarially from adult female mites to larval progeny [49]. However, past human challenge studies have demonstrated that this species is not anthropophilic [50]. It will be important to determine if the presence of *L. arenicola* and *L. deliense* in sympatry, as in Johor, increases the risk of scrub typhus to humans through maintenance of infection in small mammals by *L. arenicola* coupled with the anthropophilic habits of *L. deliense*. Notably, the absence of any negative association between these two species suggests they were not competing significantly at the Johor site. However, *W. oudemansi* exhibited negative associations with both *L. deliense* and *A. indica* in Johor and positive associations with these species in Perak, indicating context-specific competition that may be driven by the different levels of chigger diversity and abundance at each site. Differences in host composition between states might also modulate chigger interspecific competition; for instance, a study in Iran showed that the preferred “parasitope” (predilection site) on the host varied significantly between chigger species [51]. Thus, the abundance and size of certain hosts in conjunction with the preferred parasitopes of the local chigger community could affect the degree of competition in particular habitats and seasons.

Few previous chigger ecology studies have measured temperature and humidity directly during sampling, especially in the tropics. One study in the Penghu Islands measured these variables across three chigger-infested sites but no significant differences were noted between them over time [52]. However, two studies on *Eutrombicula alfreddugesi* in forest habitats, either as questing larvae in the USA (Nebraska) [53] or feeding on lizards in Chile [54], concurred that larval abundance was greatest in habitats with moderate temperatures and high relative humidity, as measured locally. To the best of our knowledge, our study is the first to show a positive, albeit weak, correlation between CSR and relative humidity. The lack of any relationship between temperature and either CSR or abundance was unexpected; although unlike the previous studies, we only measured temperature at approximately 1 m from the ground rather than at the soil level.

At provincial or national scales, human scrub typhus cases in Thailand are positively associated with humidity [13] and temperature [14], but while these variables are of key relevance to chigger biology, it is unclear if the trends in human cases are driven primarily by vector dynamics. A recent study from northern Thailand showed a strong effect of seasonal transition (especially the end of the dry season) on *Orientia* positivity in chiggers and small mammals but the pathogen was present all year round, with human cases peaking in the mid-wet season [16]. This suggests human behaviour could be the key determinant of disease risk. However, scrub typhus CSR displayed a strong positive correlation with human scrub typhus in Thailand, while a weaker negative correlation was apparent with chigger–host network connectance [15]. Although these correlations do not imply causality, they suggest that the ecology of chigger–host interactions modulates disease risk to humans, perhaps by enabling recombination between *O. tsutsugamushi* strains ahead of the main period of farming activity [55]. In our study, we did not include seasonal transition periods but the higher CSR in the dry season is consistent with our prior work in Thailand [15]. The effects of seasonality on chigger–host networks (linkages and V-ratio) was much stronger in Perak than Johor, probably because the ratio of average precipitation between the wettest and driest months is greater in the former (2.25, WMO [56]) than the latter (1.75, WMO [57]). 

The role of specific small-mammal populations and habitat types in scrub typhus ecology and the risk to humans remains especially challenging to resolve, highlighting either methodological differences between studies, a high degree of local contingency, or a combination of both. A wild host study from Thailand conducted across three provinces observed a strong association between *O. tsutsugamushi* infection and forest cover [17], and a subsequent probabilistic mapping analysis from Chiang Rai reported similar findings, as well as a link between chigger abundance and *O. tsutsugamushi* infection with the size of the host population in the previous year [18]. Conversely, a GLM analysis performed on data from the same province concluded that the rate of small-mammal (and chigger) infection with *O. tsutsugamushi* was lowest in the forest compared with three other habitat types [16]. A key recent study from Bangkok revealed an absence of *O. tsutsugamushi* in chiggers and rodents across seven parks in the city, despite the presence of *L. deliense* at four sites [47]. For human scrub typhus cases, forest cover was the single largest negative explanatory variable in Chiang Rai [14], whereas the disease was associated with forested upland habitats in Nan province [58]. However, habitat complexity was positively associated with disease incidence in Chiang Rai [14], suggesting ecotones per se, but not specific habitats, are the main drivers of disease maintenance or emergence.

In the current study, no significant associations between habitat type and infection in small mammals were detected, perhaps because of the small scale of the field sites; although in accordance with prior data from Thailand [15], CSR was greatest near the forest. Moreover, the CSR, abundance, and *Orientia* status of small mammals were not affected by host species or maturity in Malaysia, despite a marked effect of these host characteristics on CSR in Thailand [15].

## 5. Conclusions

In conclusion, our data highlight that oil palm plantations and the surrounding habitats in Malaysia are high-risk foci for scrub typhus, as they are for several other zoonotic diseases [59], engendering substantial chigger indices for small mammals. Due to increased vector abundance in the dry season, the risk of chigger bites and scrub typhus is likely to be higher at that time of year, and plantations bordering forest may support particularly large vector populations. Reducing small-mammal populations and relative humidity near the ground level (e.g., by grass cutting) are possible options to manage scrub typhus risk to plantation workers and Orang Asli living near these sites [22]. Further work in Malaysia is warranted to understand the fine-scale dynamics of *O. tsutsugamushi* in chigger–host networks in anthropized habitats across space and seasons, especially as our study was underpowered in the rice paddy, forest border, and rubbish dumpsite (probably because these habitats were not matched between states). Moreover, high-quality data on human scrub typhus incidence across Malaysia are urgently needed. Future studies using standardised methodologies, including habitat classifications across multiple scrub typhus-endemic sites, ideally in different countries, will be required before the epidemiology of this enigmatic disease is understood in proportion to its increasing importance. 

## Figures and Tables

**Table 1:** Chiggers identified and prevalence of infestation.

Species	Number Identified	Frequency (%) *	Prevalence of Infestation (%) ^^^	Notes ^~^
*Ascoschoengastia indica*	691	35.4	42.9	
*Leptotrombidium deliense*	566	29.0	43.8	
*Walchiella oudemansi*	225	11.5	23.5	
*Leptotrombidium arenicola*	157	8.0	14.8	New record on *R. exulans, T. glis*
*Gahrliepia fletcheri*	128	6.6	6.0	
*Walchia lewthwaitei*	116	5.9	8.8	New record on *T. glis*
*Eutrombicula wichmanni*	22	1.1	3.2	New record on *R. tiomanicus*
*Walchia ewingi ewingi*	16	0.8	1.8	New record on *R. tiomanicus*
*Walchia disparunguis pingue*	14	0.7	2.8	
*Walchia rustica*	11	0.6	0.9	
*Gahrliepia rutila*	3	0.2	0.5	
*Trombiculindus paniculatum*	2	0.1	0.5	New record for Malaysia
*Walchia kritochaeta*	2	0.1	0.5	New record for Malaysia
*Leptotrombidium* sp.	1	0.1	0.5	

Note: *, as proportion of identified chiggers; ^, as proportion of all hosts; ~, with reference to (Stekolnikov, 2021).

## Data Availability

The rodent (*Rattus* sp.) COI sequences generated in this study were deposited in the Barcode of Life Data System (BOLD) database and the process IDs are provided in Table S1. The *Orientia*-specific *tsa47* gene amplicon sequences generated in this study were deposited in NCBI Genbank with accession numbers OP413403 – OP413427.

## Supplementary Materials

The following supporting information can be downloaded at: www.mdpi.com/article/10.3390/pathogens11101087/s1, Figure S1: Model-average importance of terms of independent variables explaining chigger abundance and species richness; Figure S2: Unipartite network models at host individual levels by state and season; Table S1: Process IDs for *Rattus* sp. *coi* barcodes deposited in the Barcode of Life Data System (BOLD) database; Table S2: Small mammals captured at different habitat types; Table S3: Chigger infestation status based on animal host species, sex and maturity; Table S4: Number of small mammals with the indicated chigger species richness; Table S5: Number of each small-mammal species with chigger co-infestation; Table S6: Significant variables from best selected model for explaining chigger abundance; Table S7: Significant variables from best selected model for explaining chigger species richness; Table S8: Linkage density and V-ratio based on sampling site and season; Table S9: Prevalence of *Orientia tsutsugamushi* in small mammals from the Johor and Perak sites.
