# Supplementary material for: Habitat and Season Drive Chigger Mite Diversity and Abundance on Small Mammals in Peninsular Malaysia
Source: Pathogens. 2022 Sep 23;11(10):1087. doi: 10.3390/pathogens11101087 (PMC9607564; doi:10.3390/pathogens11101087)
Supplement: Supplementary file 1 [file pathogens-11-01087-s001.zip › pathogens-1906383-supplementary.pdf]

| Table of Contents | Page number |
|---|---|
| Table S1. Process IDs for *Rattus* sp. *coi* barcodes deposited in the Barcode of Life Data System (BOLD) database | 1 |
| Table S2. Small mammals captured at different habitat types | 2 |
| Table S3. Chigger infestation status based on animal host species, sex and maturity | 3 |
| Table S4. Number of small mammals with the indicated chigger species richness | 4 |
| Table S5. Number of each small mammal species with chigger coinfestation | 4 |
| Figure S1. Model-average importance of terms of independent variables explaining chigger abundance and species richness | 5 |
| Table S6. Significant variables from best selected model for explaining chigger abundance | 6 |
| Table S7. Significant variables from best selected model for explaining chigger species richness | 6 |
| Figure S2. Unipartite network models at host individual levels by state and season | 7 |
| Table S8. Linkage density and V-ratio based on sampling site and season | 8 |
| Table S9. Prevalence of *Orientia tsutsugamushi* in small mammals from the Johor and Perak sites | 8 |

**Table S1. Process IDs for *Rattus* sp. *coi* barcodes deposited the Barcode of Life Data System (BOLD) database**

| Sampling site | Process ID |
|---|---|
| Johor | UMNPA004-20 – UMNPA056-20 |
| | UMNPA058-20 – UMNPA068-20 |
| Perak | UMNPA069-20 |
| | UMNPA071-20 – UMNPA076-20 |
| | UMNPA078-20 – UMNPA080-20 |
| | UMNPA082-20 – UMNPA083-20 |
| | UMNPA085-20 |
| | UMNPA087-20 – UMNPA091-20 |
| | UMNPA093-20 – UMNPA102-20 |
| | UMNPA161-20 – UMNPA194-20 |
| | UMNPA196-20 – UMNPA216-20 |
| | UMNPA218-20 – UMNPA223-20 |

**Table S2. Small mammals captured at different habitat types**

| Habitat type | n | Animal | n |
|---|---|---|---|
| Plantation | 121 | *Rattus tanezumi* R3 mitotype | 78 |
| | | *Tupaia glis* | 21 |
| | | *Rattus tiomanicus* | 12 |
| | | *Rattus exulans* | 7 |
| | | *Rattus argentiventer* | 3 |
| Human dwelling | 47 | *Rattus tanezumi* R3 mitotype | 26 |
| | | *Tupaia glis* | 13 |
| | | *Rattus tiomanicus* | 6 |
| | | *Rattus exulans* | 2 |
| Paddy field | 26 | *Rattus argentiventer* | 22 |
| | | *Rattus exulans* | 2 |
| | | *Rattus tanezumi* R3 mitotype | 1 |
| | | *Rattus tanezumi* s.s. | 1 |
| Rubbish dumpsite | 13 | *Rattus tanezumi* R3 mitotype | 10 |
| | | *Tupaia glis* | 2 |
| | | *Rattus exulans* | 1 |
| Forest border | 10 | *Rattus tiomanicus* | 4 |
| | | *Tupaia glis* | 4 |
| | | *Rattus tanezumi* R3 mitotype | 1 |
| | | *Rattus exulans* | 1 |

**Table S3. Chigger infestation status based on animal host species, sex and maturity**

| Host species | Infestation rate | Mean chigger intensity | Median chigger intensity | Range |
|---|---|---|---|---|
| *Rattus tanezumi* R3 mitotype | 99/116 (85%) | 282 | 192 | 5-1,511 |
| *Tupaia glis* | 36/40 (90%) | 238 | 94 | 2-2,735 |
| *Rattus exulans* | 8/13 (61%) | 201 | 139 | 17-746 |
| *Rattus tiomanicus* | 20/22 (91%) | 117 | 80 | 3-477 |
| *Rattus argentiventer* | 5/25 (20%) | 63 | 27 | 1-156 |

| Host gender | Infestation rate | Mean chigger intensity | Median chigger intensity | Range |
|---|---|---|---|---|
| Female | 82/103 (80%) | 278 | 147 | 1-2,735 |
| Male | 87/114 (76%) | 206 | 141 | 2-975 |

| Host maturity | Infestation rate | Mean chigger intensity | Median chigger intensity | Range |
|---|---|---|---|---|
| Adult | 119/149 (80%) | 274 | 146 | 1-2,735 |
| Juvenile* | 48/65 (74%) | 164 | 127 | 6-731 |

| Season | Infestation rate | Mean chigger intensity | Median chigger intensity | Range |
|---|---|---|---|---|
| Dry | 118/125 (94%) | 292 | 175 | 3-2,735 |
| Wet | 51/92 (55%) | 124 | 112 | 1-588 |

*Sub-adults and juveniles were combined into a single category for this analysis

**Table S4. Number of small mammals with the indicated chigger species richness**

| Chigger species richness | Number | Percentage (%)* |
|---|---|---|
| 1 | 116 | 53.5 |
| 2 | 46 | 21.2 |
| 3 | 40 | 18.3 |
| 4 | 14 | 6.5 |
| 5 | 1 | 0.5 |

*Out of total number of small mammals (n=217)

**Table S5. Number of each small mammal species with chigger coinfestation**

| Host species | Total | Number coinfested | Percentage (%) |
|---|---|---|---|
| *Rattus argentiventer* | 25 | 0 | 0 |
| *Rattus exulans* | 13 | 7 | 53.8 |
| *Rattus phylogenetic R3* | 116 | 66 | 56.9 |
| *Rattus tanezumi* | 1 | 0 | 0 |
| *Rattus tiomanicus* | 22 | 11 | 50.0 |
| *Tupaia glis* | 40 | 17 | 42.5 |

A

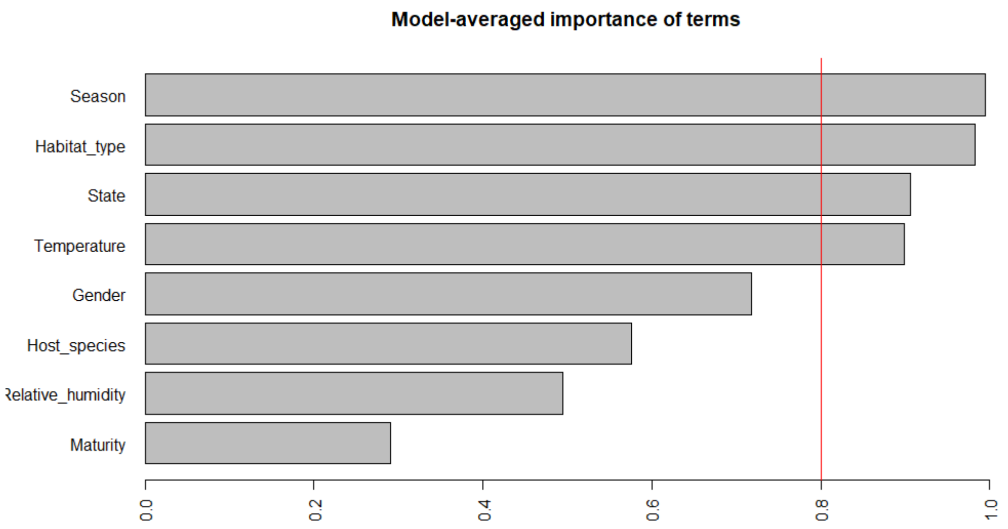


B

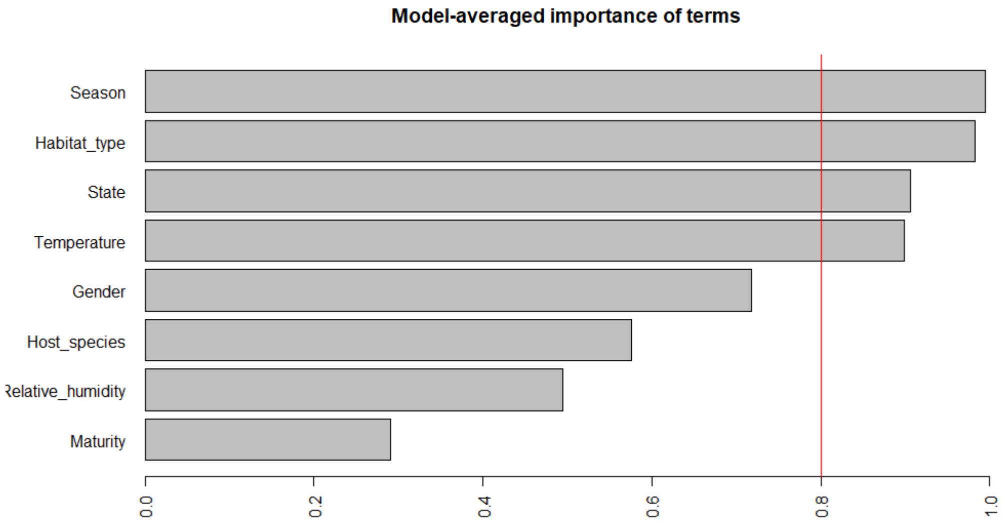


Figure S1. Model-average importance of terms of independent variables explaining chigger (a) abundance and (b) species richness. The variables with an importance score of >80% proportion support (x-axis, default in the "gmulti" package in R) were included in the final model.

**Table S6. Significant variables from best selected model for explaning chigger abundance**

| Parameters/effects | Log ratio Chi-square | P-value | Variance inflation factor |
|---|---|---|---|
| Habitat_type*** | 67.220 | <0.001 | 1.529 |
| Temperature | 0 | 0.9845 | 1.075 |
| Season*** | 21.743 | <0.001 | 1.112 |
| State* | 4.969 | 0.0258 | 1.313 |
| **Observation** | | | 216 |
| **Log-likelihood** | | | -1191.987 |
| ***AICc*** | | | 2400 |
| **The Goodness of Fit/Coefficient of determination ($R^2$)** | | | 0.9999761 |

**Table S7. Significant variables from best selected model for explaining chigger species richness**

| Parameters/effects | Log ratio Chi-square | P-value | Variance inflation factor |
|---|---|---|---|
| Habitat_type*** | 56.053 | <0.001 | 1.543 |
| Relative_humidity* | 5.341 | 0.0208 | 1.230 |
| Season*** | 35.594 | <0.001 | 1.093 |
| State*** | 11.933 | <0.001 | 1.286 |
| **Observation** | | | 216 |
| **Log-likelihood** | | | -285.057 |
| ***AICc*** | | | 586.115 |
| **The Goodness of Fit/Coefficient of determination ($R^2$)** | | | 0.9999894 |

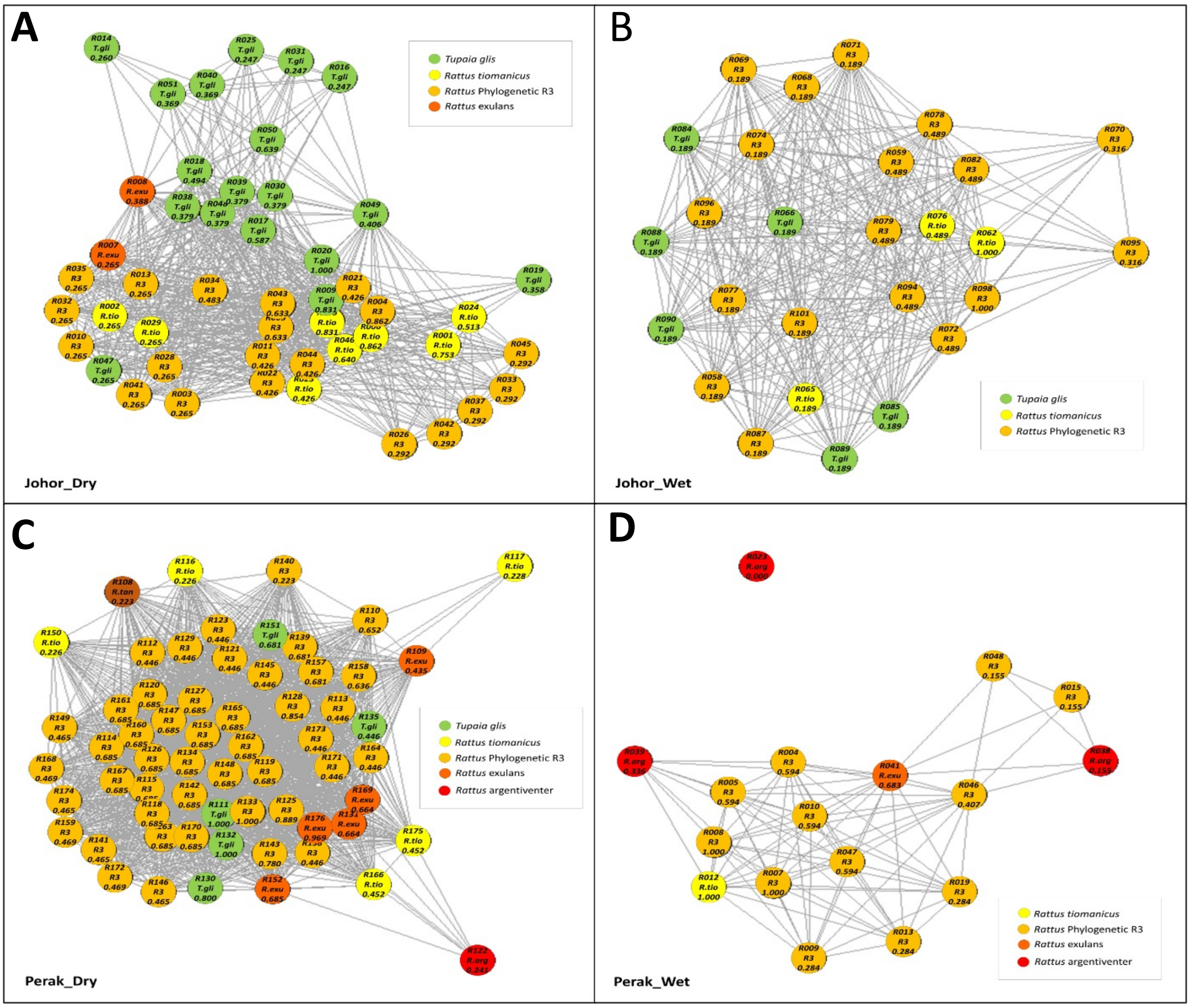


Figure S2. Unipartite network models at host individual levels by state and season. (a, b) Johor. (c, d) Perak. (a, c) Dry season. (b, d) Wet season.

**Table S8. Linkage density and V-ratio based on sampling site and season**

| Network parameters | Johor/Dry (n = 48) | Johor/Wet (n = 27) | Perak/Dry (n = 62) | Perak/Wet (n = 17) |
|---|---|---|---|---|
| Linkage density | 10.9255 | 10.7105 | 21.6172 | 5.2812 |
| V-ratio (chigger-host) | 12.3597 | 16.1200 | 33.9133 | 5.4729 |

Bipartite network parameters were calculated using 'bipartite' package in R freeware.

**Table S9. Prevalence of *Orientia tsutsugamushi* in small mammals from the Johor and Perak sites**

| Category | n | Number of *Orientia* infection | Prevalence of infection (%) | [95% CI] |
|---|---|---|---|---|
| **Host species** | | | | |
| *R. argentiventer* | 25 | 2 | 8.00 | 1.4 to 27.5 |
| *R. exulans* | 12 | 2 | 16.7 | 2.9 to 49.1 |
| *R. tanezumi R3 mitotype* | 115 | 18 | 15.7 | 9.8 to 23.9 |
| *R. tanezumi* s.s. | 1 | 0 | 0 | 0 |
| *R. tiomanicus* | 21 | 1 | 4.8 | 0.3 to 25.9 |
| *T. glis* | 40 | 2 | 5.00 | 0.9 to 18.2 |
| **Host gender** | | | | |
| Female | 102 | 13 | 12.8 | 7.2 to 21.2 |
| Male | 112 | 12 | 10.7 | 5.9 to 18.3 |
| **Maturity** | | | | |
| Adult | 149 | 18 | 12.1 | 7.5 to 18.7 |
| Sub-adult | 41 | 6 | 14.6 | 6.1 to 29.9 |
| Juvenile | 24 | 1 | 4.2 | 0.2 to 23.1 |
| **Habitat type** | | | | |
| Forest border | 9 | 0 | 0 | 0 |
| Human dwelling | 47 | 4 | 8.5 | 2.8 to 21.3 |
| Paddy field | 26 | 2 | 7.7 | 1.3 to 26.6 |
| Plantation | 119 | 18 | 15.1 | 9.5 to 23.1 |
| Rubbish dumpsite | 13 | 1 | 7.7 | 0.4 to 37.9 |
| **State** | | | | |
| Johor | 99 | 10 | 10.1 | 5.2 to 18.2 |
| Perak | 115 | 15 | 13.0 | 7.7 to 20.9 |
| **Season** | | | | |
| Dry | 123 | 14 | 11.4 | 6.6 to 18.7 |
| Wet | 91 | 11 | 12.1 | 6.5 to 21.0 |
| **Total** | **214** | **25** | **11.7** | **7.9 to 16.9** |
